# PPARδ Activation Acts Cooperatively with 3-Phosphoinositide-Dependent Protein Kinase-1 to Enhance Mammary Tumorigenesis

**DOI:** 10.1371/journal.pone.0016215

**Published:** 2011-01-13

**Authors:** Claire B. Pollock, Yuzhi Yin, Hongyan Yuan, Xiao Zeng, Sruthi King, Xin Li, Levy Kopelovich, Chris Albanese, Robert I. Glazer

**Affiliations:** 1 Department of Oncology and Lombardi Comprehensive Cancer Center, Georgetown University Medical Center, Washington, D.C., United States of America; 2 Department of Pharmacology, Georgetown University Medical Center, Washington, D.C., United States of America; 3 Department of Biostatistics, Bioinformatics and Biomathematics, Georgetown University Medical Center, Washington, D.C., United States of America; 4 Chemoprevention Agent Development and Research Group, Division of Cancer Prevention, National Cancer Institute, Bethesda, Maryland, United States of America; Institut de Génomique Fonctionnelle de Lyon, France

## Abstract

Peroxisome proliferator-activated receptorδ (PPARδ) is a transcription factor that is associated with metabolic gene regulation and inflammation. It has been implicated in tumor promotion and in the regulation of 3-phosphoinositide-dependent kinase-1 (PDK1). PDK1 is a key regulator of the AGC protein kinase family, which includes the proto-oncogene AKT/PKB implicated in several malignancies, including breast cancer. To assess the role of PDK1 in mammary tumorigenesis and its interaction with PPARδ, transgenic mice were generated in which PDK1 was expressed in mammary epithelium under the control of the MMTV enhancer/promoter region. Transgene expression increased pT308AKT and pS9GSK3β, but did not alter phosphorylation of mTOR, 4EBP1, ribosomal protein S6 and PKCα. The transgenic mammary gland also expressed higher levels of PPARδ and a gene expression profile resembling wild-type mice maintained on a diet containing the PPARδ agonist, GW501516. Both wild-type and transgenic mice treated with GW501516 exhibited accelerated rates of tumor formation that were more pronounced in transgenic animals. GW501516 treatment was accompanied by a distinct metabolic gene expression and metabolomic signature that was not present in untreated animals. GW501516-treated transgenic mice expressed higher levels of fatty acid and phospholipid metabolites than treated wild-type mice, suggesting the involvement of PDK1 in enhancing PPARδ-driven energy metabolism. These results reveal that PPARδ activation elicits a distinct metabolic and metabolomic profile in tumors that is in part related to PDK1 and AKT signaling.

## Introduction

3-Phosphoinositide-dependant protein kinase-1 (PDK1) regulates at least 23 members of the AGC protein kinase family, including AKT, p70 ribosomal S6 kinase (S6K) and all isotypes of the protein kinase C (PKC) family [1]. PDK1 primes AGC kinases by phosphorylation of a highly conserved sequence within the T-loop or activation loop [2], [3]. Although, PDK1 is constitutively active [4], [5], its activity may be further enhanced by transphosphorylation on tyrosine and serine residues by other kinases. PDK1 can also regulate cell signaling in other capacities, such as serving as a nucleo-cytoplasmic shuttling protein [6], an activator of RalGDS [7], recruiting PKCθ and scaffold protein CARD11 to lipid rafts [8], and as a co-activator of peroxisome proliferator-activated receptorγ (PPARγ) in adipogenesis [9]. PDK1 plays an important role in mediating the effects of insulin and growth factors that regulate cell proliferation, cell size, differentiation and survival [10]. While homozygous deletion of PDK1 is embryonic lethal [10], [11], hypomorphic mice with a 90% PDK1 deficiency develop normally. In contrast, PDK1 expression and gene copy number are increased in breast cancer [12], [13], [14] and breast cancer cell lines [15], which is consistent with down-regulation of PDK1 inhibiting cancer cell migration and metastases [16], and over-expression of PDK1 inducing transformation, drug resistance, invasion and tumorigenicity [13], [17], [18]. Thus, PDK1 appears to have diverse roles in both normal and malignant cells through kinase-dependent and -independent mechanisms.

PPARs belong to the nuclear receptor superfamily of ligand-dependent transcription factors, which control metabolic and inflammatory signaling associated with diabetes and lipodystrophies [19], but also regulate genes associated with proliferation, survival and angiogenesis in tumor cells [20], [21], [22]. Among the three PPAR isotypes, PPARδ is distinguished by its ability to function as a promoter of tumorigenesis in many instances [21], and is highly expressed in colon cancer [23], [24], head and neck cancer [25], endometrial cancer [26] and breast cancer [21]. PPARδ activation promotes breast and prostate cancer cell growth [20] and a more aggressive phenotype [27]. In lung cancer cells, PPARδ activation reduces PTEN to increase PDK1 and AKT activity and co-regulate their expression in concert with proliferation [28]. PPARδ agonists accelerate mammary carcinogenesis [29] and promote metastatic gastric tumorigenesis [30], whereas, disruption of PPARδ blocks mammary and colon tumorigenesis [31], [32], although studies to the contrary have been reported [33], [34]. The tumor promoting effects of PPARδ may be related in part to activation of PDK1, which is a PPARδ target gene in keratinocytes [35], [36]. PDK1 and PPARδ co-associate in DMBA-induced mammary tumors [9], [29], and PPARδ activates PI3K/PDK1 signaling in a diverse range of cell types to enhance survival and growth [36], [37], [38]. Thus, there is evidence to suggest that PDK1 and PPARδ may act cooperatively in tumorigenesis.

The *in vivo* consequences of PDK1 transgene expression in the mammary gland, its effect on tumorigenesis, and its influence on the tumor promoting effects of PPARδ activation have not been investigated. To address these questions, transgenic mice were generated that express PDK1 in the mammary gland under the control of the mouse mammary tumor virus (MMTV) enhancer/promoter sequence in the long terminal repeat. Here we report that PDK1 transgene expression induced PPARδ and elicited a PPARδ-like gene expression profile associated with glucose and lipid metabolism. Treatment with the selective PPARδ agonist GW501516 markedly accelerated mammary carcinogenesis, particularly in MMTV-PDK1 mice, and the reduction in tumor latency correlated with a metabolic gene expression and metabolomic signature that differed from wild-type animals. These results suggest that the tumor promoting effects of a PPARδ agonist are associated in part with PDK1 and a distinct metabolic profile related to glycolysis utilization and lipid biosynthesis.

## Materials and Methods

MMTV-PDK1 mice were generated by pronuclear injection of FVB mouse embryos as previously described [39]. The N-terminal Myc-tagged human PDK1 cDNA [4] was kindly provided by Dr. Dario Alessi, University of Dundee, and cloned into the *Eco*R1 site in the MMTV-SV40-Bssk vector provided by Dr. William Muller, McMaster University, Hamilton, Ontario, Canada. The MMTV-PDK1 construct was digested with *Sal* I-*Spe* I, purified and used for microinjection. All animal studies were conducted under protocols 07-017 and 09-061, “Chemopreventive agents in mammary progenitor cell targeting in carcinogen-induced breast cancer”, approved by the Georgetown University Animal Care and Use Committee in accordance with NIH guidelines for the ethical treatment of animals.

### Genotyping

Primers designed to detect a fragment unique to the MMTV-PDK1 transgene were: forward: 5′ CGC CGC AGC CTC GGA AGA AGC GGC, reverse: 5′GGG TAC CTC ACT GCA CAG CGG CGT CC. Mice were screened for transgene expression by PCR using tail DNA.

### Western blot analysis

Lysates were prepared from either tumor cell cultures or mammary glands using a lysis buffer containing: 50 mM Tris-HCl (pH 7.5), 0.5% NP-40, 0.1% SDS, 0.25% sodium deoxycholate, 125 mM NaCl, 1 mM EDTA, 50 mM NaF, 1 mM sodium orthovanadate, 2.5 mM sodium pyrophosphate, 1 mM sodium β-glycerophosphate, 1 mM PMSF, and a protease inhibitor cocktail (Roche Molecular Biochemicals). Mammary tissue was snap-frozen in liquid nitrogen and powdered in a mortar and pestle. Following incubation on ice for 30 min, lysates were cleared by centrifugation at 13,000×g for 15 min at 4°C. Protein concentration was determined using the Coomassie Plus Protein Assay (Pierce), and 20–50 µg of lysate were separated in a 4–12% NuPAGE Bis-Tris gel (Invitrogen). After wet transfer, membranes were blocked for 5 min at room temperature in 0.5% non-fat dry milk in Tris-buffered saline containing 0.1% Tween-20. Primary antibody was incubated for either 1.5 hr at room temperature or overnight at 4°C. Secondary antibody was incubated for 30 min at room temperature, and proteins were visualized with West Pico Stable (Pierce). Antibodies and their dilution were the following: AKT1 and PDK1 (1∶2,000, Upstate Biotechnology), β-actin (1∶5,000, Sigma-Aldrich), pS241PDK1 and pS308AKT, PKCα and pS657PKCα (1∶1,000, Cell Signaling Technologies), PTEN (1∶1,000, Santa Cruz Biotechnology), PPARδ (1∶1,000, Zymed) and horseradish peroxidase-conjugated anti-rabbit IgG and anti-mouse IgG (1∶5,000, Pierce).

### Mammary carcinogenesis

DMBA (dimethylbenz(*a*)anthracene, Sigma) was dissolved in cottonseed oil at a concentration of 10 mg/ml. MMTV-PDK1 mice and wild-type littermates from founder line 192 were administered medroxyprogesterone/DMBA as previously described [40]. Briefly, 5 week-old virgin female mice were injected s.c. with 15 mg of medroxyprogesterone acetate suspension (Sicor Pharmaceuticals, Inc.), and one week later were administered 4 weekly doses of 1 mg DMBA in 0.1 ml cottonseed oil by gavage. A diet supplemented with 0.005% GW501516 was started one day after the last dose of DMBA [29]. GW501516 was provided by the Chemoprevention Branch, National Cancer Institute. Mice were euthanized by carbon dioxide inhalation when tumors reached 1–1.5 cm^3^. All animal protocols were approved by the Georgetown University Animal Care and Use Committee.

### Histopathology

Tumor samples were dissected free of connective tissue and fixed in formalin. Paraffin blocks were prepared for hematoxylin & eosin (H&E) staining by the Histopathology and Tissue Shared Resource, Lombardi Comprehensive Cancer Center (LCCC), Georgetown University. Tumors were classified using the histological nomenclature recommended by Cardiff et al. [41] as adenocarcinomas, including acinar and solid lobular types, adenosquamous and squamous carcinomas and myoepithelial and undifferentiated carcinomas as previously described [40].

### Immunohistochemistry

IHC analysis was carried out as previously described [29], [39], [40]. The following primary antibodies were: mouse anti-PDK1 (1∶50, sc-17765, Santa Cruz Biotechnology), rabbit anti-pT308AKT (1∶600, sc-16646, Santa Cruz Biotechnology), rabbit anti-PPARδ (1∶200, sc-7197, Santa Cruz Biotechnology).

### Gene Microarray

Mammary gland tissue was excised and preserved in RNAlater (Ambion) at −20°C until RNA was extracted using an RNeasy Mini Kit (Qiagen) according to the manufacturer's protocol. RNA purity was assessed by an A_260_/A_280_ ratio of ≥1.9, and RNA quality was monitored using a microfluidic nanochip (Agilent). cRNA synthesis was carried out using the Affymetrix protocol with minor modifications as previously described [40]. Biotin-labeled cRNA was fragmented at 94°C for 35 min and used for hybridization overnight to an Affymetrix mouse 430A2.0 GeneChip® by the Genomics and Epigenomics Shared Resource, LCCC, Georgetown University. The GeneChip® was scanned using an Agilent Gene Array scanner, and grid alignment and raw data generation performed with the Affymetrix GeneChip® Operating software 1.1. A noise value (*Q*) based on the variance of low-intensity probe cells was used to calculate a minimum threshold for each GeneChip. Data generated after scanning was subjected to comparison analysis to select change calls at 100% increase or decrease compared with control for each gene. Gene array analysis was further refined by evaluating differences between paired samples and ranking changes by their log_2_ ratio. Differences in signal ratio >log_2_ 2.0 and <log_2_ −2.0 were ranked, and genes with a signal of <300 in all experimental groups were excluded from analysis. Each cRNA was prepared from equal amounts of RNA pooled from 5 mice per group. Hierarchical clustering was carried out by standardizing transformed log base 2 values from the raw intensities using the equation 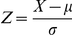, and multiplying by a constant to adjust all values from −3 to +3. Euclidean distances, as described in 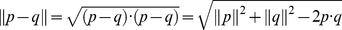 were used to compute distances between genes as well as samples separately. Finally the average linkage as 
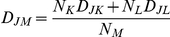
 was applied to determine the gene clusters and sample clusters. All data is MIAME compliant and the raw data has been deposited in the GEO database.

### Metabolomic analysis

Metabolomic analysis was performed using ultraperformance liquid chromatography electrospray ionization time-of-flight mass spectrometry (UPLC-ESI-TOFMS) [42] by the Proteomic and Metabolomics Shared Resource, LCCC, Georgetown University. Experimental groups consisted of mammary glands from each of five control wild-type and transgenic littermates and an equal number treated with GW501516 for seven days. Tissue was snap-frozen in liquid nitrogen and stored at −80°C. Extracts were prepared in 50% methanol containing internal standards using MagNAlyser Green Beads and a MagNA Lyser agitator (Roche). Samples were clarified, and processing and multivariate analysis performed as described [42]. Each sample (5 µl) was injected onto a reverse-phase 50×2.1 mm ACQUITY® 1.7-µm C18 column (Waters Corp, Milford, MA) using an ACQUITY® UPLC system (Waters) with a gradient mobile phase consisting of 2% acetonitrile in water containing 0.1% formic acid (A) and 2% water in acetonitrile containing 0.1% formic acid (B). Each sample was resolved for 10 min at a flow rate of 0.5 ml/min. The gradient consisted of 100% A for 0.5 min then a ramp of curve 6 to 100% B from 0.5 min to 10 min. The column eluent was introduced directly into the mass spectrometer by electrospray. Mass spectrometry was performed on a Q-TOF Premier® (Waters) operating in either negative-ion (ESI−) or positive-ion (ESI+) electrospray ionization mode with a capillary voltage of 3200 V and a sampling cone voltage of 20 V in negative mode and 35 V in positive mode. The desolvation gas flow was set to 800 liters/h and the temperature was set to 350°C. The cone gas flow was 25 liters/hr, and the source temperature was 120°C. Accurate mass was maintained by introduction of LockSpray® interface of sulfadimethoxine (311.0814 [M+H]^+^ or 309.0658 [M−H]^−^) at a concentration of 250 pg/µl in 50% aqueous acetonitrile and a rate of 150 µl/min. Data were acquired in centroid mode from 50 to 850 *m/z* in MS scanning. Centroided and integrated mass spectrometry data from the UPLC-TOFMS were processed to generate a multivariate data matrix using MarkerLynx® (Water) that was used for analysis by SIMCA-P+11 software (Umetrics), and classified with Random Forest. Principal components analysis (PCA) and partial least-squares discrimination analysis (PLS-DA) was performed on Pareto-scaled MarkerLynx matrices to identify candidate metabolites that distinguished WT from transgenic tissue, as well as tissues from animals treated with GW501516. Metabolites were identified using the Madison Metabolomics Database Consortium, Lipidmaps and Scripps Centre for Mass Spectrometry, and negative-ion and positive-ion electrospray mode. Metabolites were verified using tandem MS by comparison to authentic compounds.

### Cell culture and reporter gene analysis

Comma-1D/SRα and Comma-1D/PDK1 cells [15], [43] were grown in 24-well plates in DMEM medium containing 5% fetal calf serum. After 24 hr, medium was replaced with DMEM-containing 5% stripped FBS (Invitrogen). Cells were transfected using Lipofectamine Plus (Invitrogen) and the reporter plasmids pTopFlash (Invitrogen) or pGL2–3XPPRE–Luciferase reporter plasmid (provided by Dr. Mitchell Lazar, University of Pennsylvania) together with either control pcDNA3.1 or pcDNA3.1 expressing dominant-negative PPARδ (mPPARδR112P) (Y. Yin and R.I. Glazer, unpublished results). PPRE-luciferase activity was measured in the presence of GW501516, and pTopFlash was used to measure β-catenin/TCF-dependent transcription. Luciferase activity was measured with the Dual-Luciferase assay system (Promega, Madison, WI).

### Statistical Analysis

Tumor survival data were assessed by Kaplan-Meier analysis by the log rank test, and tumor number incidence by the Mann-Whitney U test using GraphPad Prism version 4 (GraphPad Software). Histological analysis of tumors was performed by Fishers' Exact test. Differences were considered to be significant at *P*<0.05.

## Results

### MMTV-PDK1 transgenic mice

**Figure 1:**
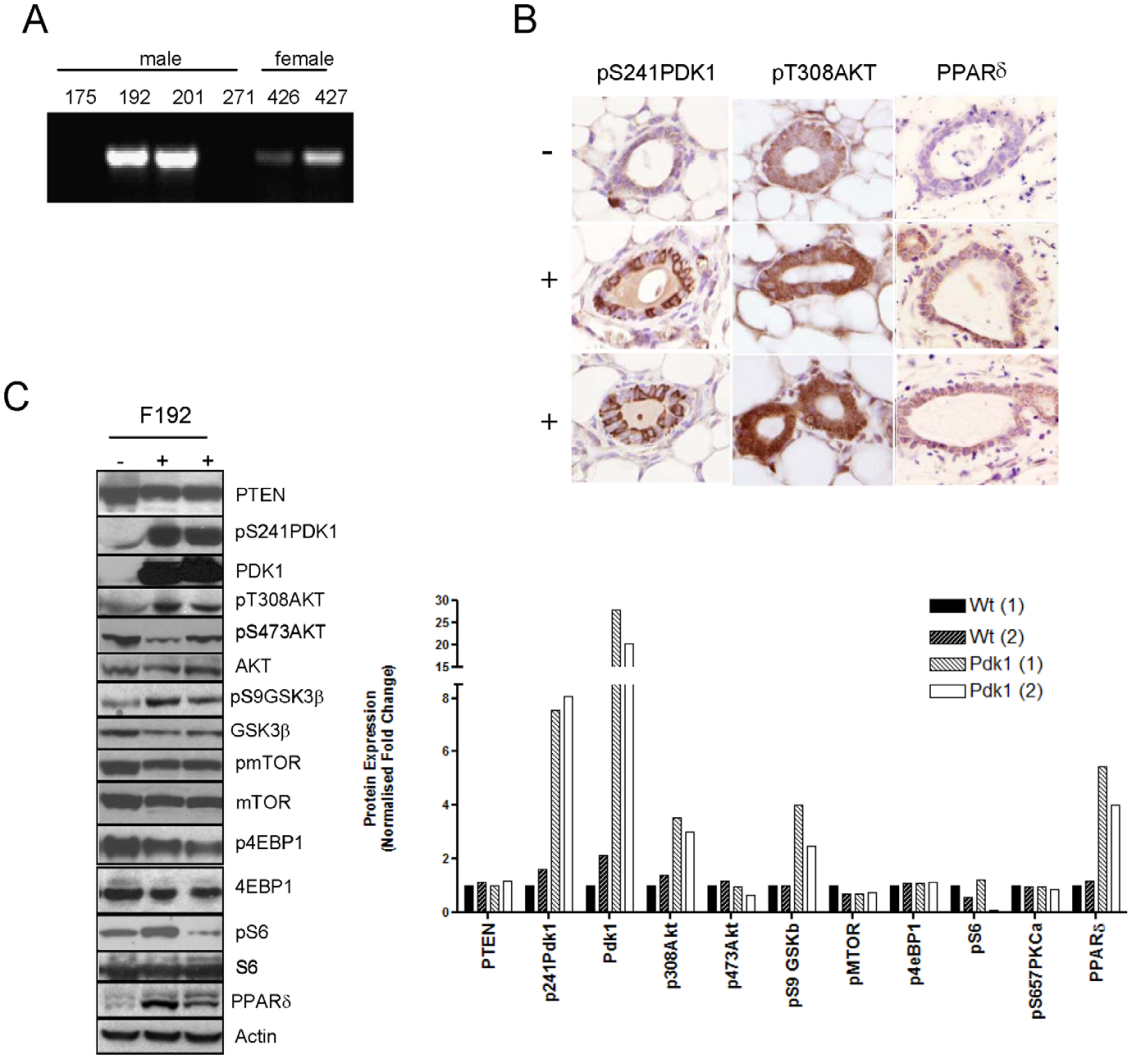
PDK1 transgene expression and analysis of downstream signaling. (A) PCR analysis. DNA was prepared from tail DNA and transgene expression analyzed by PCR using primers specific for the MMTV-PDK1 transgene. The results indicate that MMTV-PDK1 is expressed in four founder lines. (B) IHC analysis indicates that pS241PDK1, pT308AKT and PPARδ were increased in the mammary gland of two nulliparous MMTV-PDK1 mice (*+*) vs. a wild-type littermate (*−*) from founder line 192. Magnification 600×. (C) Western analysis. Mammary gland lysates from two 7 week-old nulliparous MMTV-PDK1 (*+*) mice and one wild-type (*−*) littermate derived from founder line 192 were analyzed by western blotting. Founder line 192 expressed increased pT308AKT, pS9GSK3β and PPARδ. The *bar graph* represents quantitation of the western blots normalized to either non-phosphorylated protein or βactin levels.

MMTV-PDK1 transgenic mice were screened for transgene expression by PCR of tail DNA, and four founder lines were identified (Figure 1A). Founder line 192 expressed 8–10-fold higher pS241PDK1 and PDK1 levels vs. wild-type littermates, compared to a 1.5–2-fold change in other founder lines (results not shown), and thus, founder 192 was used for all subsequent studies. The mammary gland of nulliparous transgenic mice exhibited normal glandular structure and ductal elongation and branching at 3 and 12 weeks of age (Supporting information, Figure S1A). Lactating transgenic mice exhibited strong PDK1 expression (Supporting information, Figure S1B), but no delay in involution vs. wild-type littermates (Supporting information, Figure S1C). There were no differences between transgenic and wild-type mice in their hyperplastic response to medroxyprogesterone stimulation, and transgenic mice did not present with mammary tumors over their lifespan (results not shown).

### MMTV-PDK1 mice express increased levels of pT308AKT, pS9 GSK3β and PPARδ

IHC analysis of the mammary gland of 7 week-old pups indicated increased pS241PDK1, pT308AKT and PPARδ expression vs. wild-type littermates (Figure 1B). Western analysis revealed that increased PDK1 transgene expression was associated with a 3–4-fold increase in pT308AKT and a 4–5.5-fold increase in PPARδ (Figure 1C), whereas, pS473AKT remained unaltered (Figure 1C), as previously reported in PDK1 null cells [44]. The levels of pS9GSK3β were increased 2.5–3.5-fold in transgenic mice, but no changes were noted in PTEN, pmTOR, p4EBP1, pS6 and pS657PKCα (Figure 1C). qRT-PCR analysis indicated that PDK1 and PPARδ mRNA levels were increased 3- and 1.4-fold, respectively (results not shown) in comparison to the large increases in protein expression, suggesting that their co-regulation is in part due to post-translational regulation.

### MMTV-PDK1 mice are sensitized to PPARδ agonist GW501516

**Figure 2:**
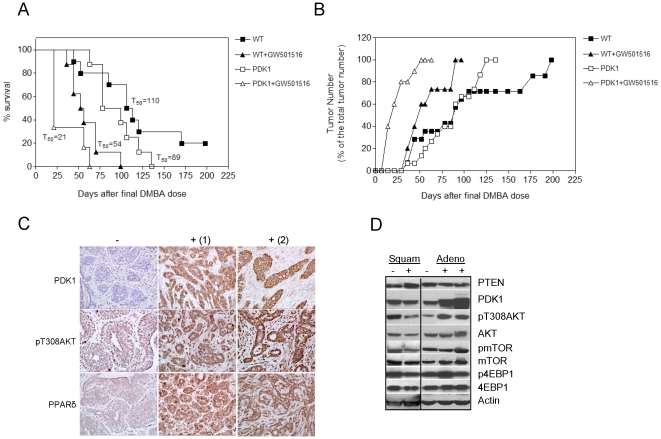
GW501516 enhances DMBA-induced mammary carcinogenesis. (A) Following medroxyprogesterone/DMBA treatment, tumor latency was similar between MMTV-PDK1 mice and wild-type littermates, but was reduced in both groups by treatment with the PPARδ agonist GW501516. Survival is defined as the time at which tumors reached ∼1000 mm^3^ and animals were euthanized. The median tumor-free interval (*T_50_*) was 89 days for MMTV-PDK1 mice (*PDK1*) vs. 110 days for wild-type mice (*WT*) (P = 0.1738); 54 days for GW501516-treated wild-type animals vs. 110 days for WT mice (P = 0.0019); 21 days for GW501516-treated PDK1 mice vs. PDK1 mice (P = 0.0002). Statistical analysis used the log rank test. The number (N) of mice per group was: WT (N = 10), WT+GW (N = 10), PDK1 (N = 8), PDK1+GW (N = 6). (B) Cumulative mammary tumor formation. The total number of tumors is expressed as a percentage of total tumor number. Percent tumor formation between GW501516-treated WT vs. WT mice (*P* = 0.0127), GW501516-treated PDK1 vs. PDK1 mice (*P* = 0.0011) and GW501516-treated PDK1 vs. WT mice (*P* = 0.0001 were statistically significant. Tumor formation in WT vs. PDK1 mice (P = 0.710) was not significantly different. (C) IHC analysis indicates that PDK1, pT308AKT and PPARδ expression are increased in two adenocarcinomas from MMTV-PDK1 mice (+) vs. an adenocarcinoma from a wild-type littermate (−). Magnification 600×. (D) Immunoblot analysis indicates that PDK1 and pT308AKT are elevated in adenocarcinomas from MMTV-PDK1 mice (+) vs. wild-type mice (−), but were unchanged in squamous carcinomas.

Since MMTV-PDK1 mice did not present with mammary tumors over their lifespan, they were tested for their susceptibility to mammary carcinogenesis induced by progestin stimulation and DMBA [40] (Figure 2A, B). MMTV-PDK1 mice did not exhibit a statistically significant change in tumor latency, where median tumor-free survival was 89 days vs. 110 days in wild-type mice (Figure 2A). However, maintaining animals on a diet containing the selective and potent PPARδ agonist, GW501516, immediately following DMBA, resulted in a dramatic acceleration of tumor formation (Figure 2A). The tumor promoting effect of GW501516 was more pronounced in MMTV-PDK1 mice, where GW501516 treatment reduced the median tumor-free survival from 89 days to 21 days. This represented a two-fold greater reduction in survival than observed in wild-type mice treated with GW501516, where the median tumor-free survival was reduced from 110 days to 54 days (Figure 2B). Tumor multiplicity was similar in both groups (1.88 and 1.40, respectively) and was not altered by GW501516 treatment (Table 1). Although there were no significant differences in tumor histopathology between PDK1 and wild-type mice, GW501516 treatment produced a significant increase in the percentage of adenosquamous and squamous cell carcinomas in both groups, which correlated with rapid tumor development (Table 1).

**Table 1:** Tumor histology and multiplicity.

		% of each histotype		
Histopathology:	WT	WT+GW*	PDK1	PDK1+GW**
Adenocarcinoma	57	13	34	0
Adenosquamous/squamous	29	80	33	91
Myoepithelial/undifferentiated	14	7	33	9
Tumor multiplicity	1.40	1.5	1.88	1.67
No. of tumors	14	15	15	10
No. of animals	10	10	8	6

*P = 0.0125 vs. untreated WT mice for histological differences.

**P = 0.0205 vs. untreated PDK1 mice for histological differences.

Wild-type (WT) and MMTV-PDK1 transgenic mice (PDK1) were fed either standard rodent chow or chow supplemented with 0.005% (w/w) GW501516 (GW). GW501516 treatment produced a significant change in the percentage of adenosquamous/squamous carcinomas. There were no significant differences in tumor multiplicity between groups.

### Adenocarcinomas from MMTV-PDK1 mice express increased pT308AKT and PPARδ

HC analysis indicated that adenocarcinomas induced in MMTV-PDK1 mice expressed elevated levels of PDK1, pT308AKT and PPARδ in comparison to histologically matched tumors from wild-type mice (Figure 2C). Western analysis confirmed that this phenotype was present in adenocarcinomas, but not in squamous cell carcinomas from transgenic mice, as might be expected from the mammary epithelium selectivity of the MMTV promoter. However, no changes were evident in other putative PDK1 downstream targets in either tumor histotype (Figure 2D).

### PPARδ activation increases PDK1 and pT308AKT

**Figure 3:**
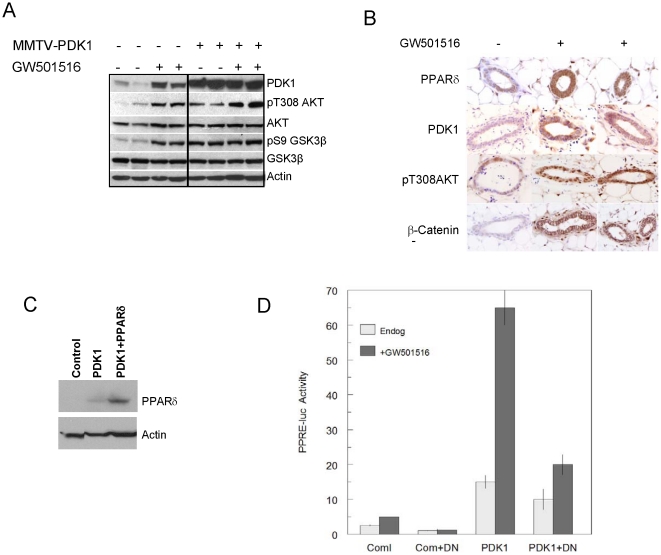
GW501516 increases PDK1, pT308AKT, PPARδ and β-catenin expression in the mammary gland. (A) Immunoblot analysis of mammary gland lysates from a wild-type (*WT*) and two MMTV-PDK1 (*PDK1*) mice maintained on either standard (−) or GW501516-supplemented (+) diets. The results indicate that GW501516 increased PDK1 and pT308AKT in both groups, and pS9GSK3β in wild-type mice. (B) IHC analysis indicates that PDK1, pT308AKT, PPARδ and β-catenin were preferentially increased in wild-type mice following GW501516 treatment. Magnification 600×. (C) Comma-1D mammary epithelial cells transduced with either empty virus (*Contro*l) or PDK1-expressing virus (*PDK1*), and assessed for PPARδ expression by western blotting. PDK1 cells transfected with PPARδ (PDK1+PPARδ) served as a positive control. PPARδ expression was increased in PDK1 cells. (D) Cells in (C) were examined for PPARδ-dependent reporter gene activity in the presence and absence of GW501516 and dominant-negative PPARδ (*DN-PPARδ*). PPARδ-dependent was increased >10-fold in PDK1-expressing cells vs. control cells, and activity was suppressed by DN-PPARδ.

To evaluate the effect of PPARδ activation on PDK1 signaling, mammary tissue was analyzed from wild-type and transgenic animals maintained on the GW501516 diet (Figure 3A). GW501516-treated wild-type mice expressed increased levels of PDK1, pT308AKT and pS9GSK3β, whereas, transgenic mice exhibited increased pT308AKT, but no additional changes in PDK1 and pS9GSK3β, a result that we attribute to their already elevated levels in the absence of GW501516 treatment. IHC analysis of wild-type mice maintained on the GW501516 diet confirmed increased levels of PDK1 and pT308AKT and PPARδ, and β-catenin although elevated, was primarily membrane-associated and not nuclear (Figure 3B).

The causal association between PDK1 over-expression and PPARδ was also investigated in mouse mammary epithelial cell line Comma-1D stably expressing PDK1 [43], which expresses high β-catenin/TCF transcriptional activity [15]. Comma-1D/PDK1 cells, but not control cells exhibited increased levels of PPARδ (Figure 3C), and reporter gene assays in the presence and absence of GW501516, as well as in the presence of dominant-negative PPARδ, confirmed that the receptor was transcriptionally active (Figure 3D). Similar studies in HCT116 cells, which also express PPARδ and high β-catenin/TCF transcriptional activity confirmed the dependence of both pathways on PDK1 (results not shown).

### MMTV-PDK1 mice express a gene and metabolomic profile reflecting PPARδ activation

To further characterize the phenotype of MMTV-PDK1 mice and the effect of GW501516 treatment, gene expression profiling was carried out with mammary tissue from transgenic and wild-type mice maintained on the GW501516 diet for 1 week (Supporting information, Figure S2, Table S1, Table S2). Hierarchical cluster analysis revealed changes in gene expression that were specifically associated with GW501516 treatment of both wild-type and transgenic mice that were related to lipid (Acaca, Acly, Elovl6, Acss2) and glucose (Acly, PDK4, Slc2a5) metabolism (Table 2, Group A, Supporting information Figure S2A, Table S2), as well as non-metabolic functions (Asb5, Hrc, Mmd2, Smpx, Tcap, Trdn) (Table 2, Group A′, Supporting information Figure S2A, Table S2). There was also an additional gene signature common to MMTV-PDK1 and GW501516-treated wild-type mice that was not associated with increased tumorigenesis (Cox7a1, Cpt1b, Elovl3, Fabp3, E2f5, Slcf5, Ucp1) (Table 2, Group B, Supporting information Figure S2A, Table S2).

**Table 2:** Gene microarray analysis: heatmap groups.

Symbol	Description	Fold	Change
**Group A**		**WT+GW vs WT**	**PDK1+GW vs WT**
Acaca	acetyl-Coenzyme A carboxylase alpha	3.4	3.0
Acss2	acyl-CoA synthetase short-chain family member 2	3.1	2.7
Acly	ATP citrate lyase	3.8	2.5
Cspg3	chondroitin sulfate proteoglycan 3	4.4	4.1
Elovl6	ELOVL family member 6, elongation of long chain fatty acids (yeast)	3.5	3.7
Hspa9a	heat shock protein 9A	3.3	2.7
Pdk4	pyruvate dehydrogenase kinase, isoenzyme 4	2.6	2.5
Slc2a5	solute carrier family 2 (facilitated glucose transporter), member 5	4.5	3.1
Tatdn1	TatD DNase domain containing 1	3.2	2.8
**Group A′**		**WT+GW vs WT**	**PDK1+GW vs WT**
Asb5	ankyrin repeat and SOCs box-containing protein 5	−1.3	−1.7
Hrc	histidine rich calcium binding protein	−1.6	−2.4
Mmd2	monocyte to macrophage differentiation-associated 2	−2.0	−1.7
Smpx	small muscle protein, X-linked	−1.8	−3.6
Tcap	titin-cap	−2.3	−5.5
Trdn	triadin	−4.5	−3.7
**Group B**		**WT+GW vs WT**	**PDK1 vs WT**
Cox7a1	cytochrome c oxidase, subunit VIIa 1	5.3	3.7
Cpt1b	carnitine palmitoyltransferase 1b, muscle	3.0	2.7
Elovl3	elongation of very long chain fatty acids (FEN1/Elo2, SUR4/Elo3, yeast)-like 3	18.2	9.4
Fabp3	fatty acid binding protein 3, muscle and heart	4.6	4.9
E2f5	E2F transcription factor 5	4.3	4.6
Slc27a2	solute carrier family 27 (fatty acid transporter), member 2	3.7	3.9
Ucp1	uncoupling protein 1 (mitochondrial, proton carrier)	6.5	4.6

Shown is the relative expression of metabolic genes common to GW510516-treated (GW) wild-type mice (WT), MMTV-PDK1 transgenic mice (PDK1) and GW501516-treated MMTV-PDK1 mice (PDK1+GW) vs. WT. The heatmap groups refer to Figure S2A, and the gene expression profile of all groups is presented in Table S1.

**Figure 4:**
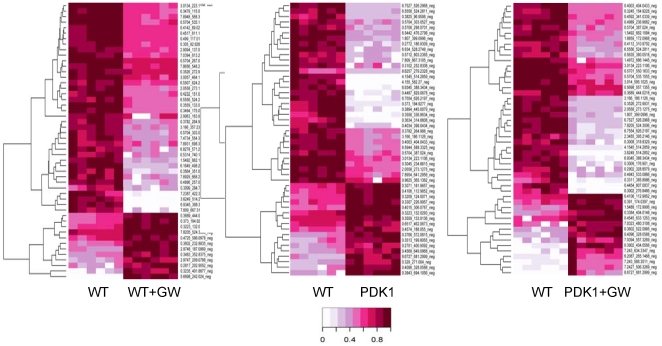
The metabolome of GW501516-treated wild-type and MMTV-PDK1 mice. Wild-type (*WT*) and MMTV-PDK1 (*PDK1*) mice were maintained for 7 days on either standard or GW501516 (*GW*)-supplemented diets. The metabolite levels in mammary gland extracts were analyzed by UPLC-ESI-TOFMS. The heatmaps depict the changes in negative ions for control and GW-treated WT and PDK1 mice in 6 replicate analyses.

Metabolomic analysis of the mammary gland revealed similarities and differences between the metabolome of GW501516-treated wild-type and transgenic animals, as well as between control wild-type and transgenic mice. (Figure 4, Table 3, Supporting information Figure S4, Table S3, Table S4, Table S5). The suggestion of increased lipid biosynthesis based on gene array data in GW501516-treated animals was consistent with increased fatty acid and phospholipid levels, and correlated with increased tumorigenesis (Table 3), despite the differences in specific metabolites between the two groups. In contrast, untreated transgenic mice exhibited a reduction in lipid metabolites vs. wild-type mice.

**Table 3:** Metabolomic profile in MMTV-PDK1 and GW501516-treated animals.

MS Mass	CQ_ID	Name	Fold Change	Value		Algorithm Identifer	ESI
				**WT**	**WT+GW**		
422.3301	cq_20090	Linoleyl carnitine	−13.3	0.0041	0.0003	RF	[M−H]
788.5236	cq_19896	Diarachidonoylphosphatidylethanolamine	41.0	0.0001	0.0023	SIMCA	[M+H]
				**WT**	**PDK1**		
216.0640	cq_00846	sn-Glycero-3-phosphoethanolamine	−4.0	0.0017	0.0004	SIMCA	[M+H]
280.2402303	cq_20055	Linoelaidic acid; 9E,12E-Octadecadienoic acid	−2.3	0.0162	0.0071	RF	[M+H]
388.2614	cq_10636	7a-Hydroxy-3-oxo-4-cholenoic acid	−4.7	0.0021	0.0004	RF	[M+H]
428.3725	cq_10816	Stearoylcarnitine	−1.9	0.0116	0.0060	SIMCA	[M+H]
450.2620	cq_18534	1–16:1-Lysophosphatidylethanolamine	−6.7	0.0128	0.0019	SIMCA	[M−H]
452.2787	cq_18660	1–16:0-Lysophosphatidylethanolamine	−1.6	0.0443	0.0283	SIMCA	[M−H]
478.2954	cq_18657	1–18:1-Lysophosphatidylethanolamine	−6.0	0.0309	0.0052	SIMCA	[M−H]
				**WT**	**PDK1+GW**		
279.2328	cq_20055	Linoelaidic acid; 9E,12E-Octadecadienoic acid	4.4	0.0071	0.0313	RF	[M−H]
303.2321	cq_17815	Arachidonic acid; Eicosatetraenoic acid	4.2	0.0080	0.0336	SIMCA	[M−H]
303.2321	cq_17815	Arachidonic acid; Eicosatetraenoic acid	2.5	0.0108	0.0272	RF	[M+H]
424.3413	cq_20090	Linoleyl carnitine	2.6	0.0041	0.0106	SIMCA	[M+H]
450.2620	cq_18534	Palmitoleic acid; 9Z-Hexadecenoic acid	14.3	0.0003	0.0043	SIMCA	[M−H]
450.2620	cq_18534	1–16:1-Lysophosphatidylethanolamine	12.1	0.0010	0.0117	SIMCA, RF	[M−H]
454.2913	cq_18660	1–16:0-Lysophosphatidylethanolamine	2.0	0.0089	0.0178	SIMCA	[M+H]
480.3073	cq_18657	1–18:1-Lysophosphatidylglycerol	2.5	0.0080	0.0202	SIMCA, RF	[M+H]
507.2736	cq_18985	1–18:2-Lysophosphatidylglycerol	2.2	0.0034	0.0076	SIMCA	[M−H]

RF, Random Forest.

## Discussion

**Figure 5:**
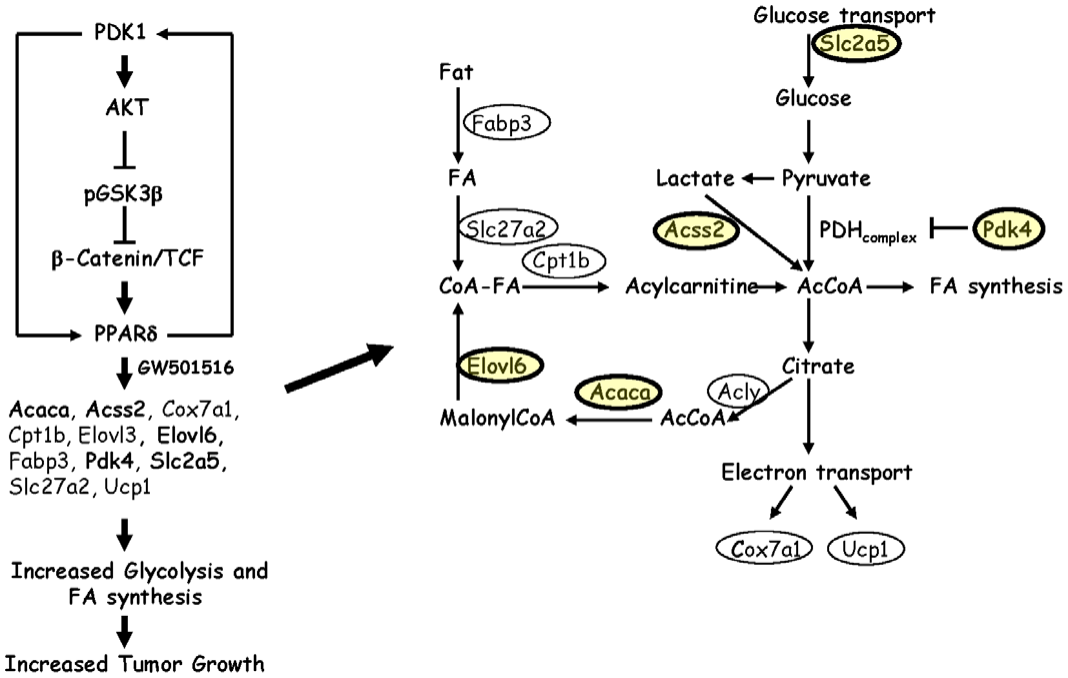
Schematic of the regulation of glucose and fatty acid metabolism by PDK1 and PPARδ. One mechanism depicted is that PPARδ upregulates PDK1 expression and activates AKT to inhibit GSK3β and increase β-catenin/TCF-dependent transcription; however, it is controversial whether PPARδ is a TCF target gene. Activation of PPARδ by GW501516 in wild-type and MMTV-PDK1 mice upregulates the expression of genes (highlighted in *yellow*) associated with glucose transport (Slc2a5), acetylCoA formation through acetylCoA (Acss32, Pdk4), and fatty acid biosynthesis (Acaca, Elovl6). Genes associated with fatty acid transport and transcriptional regulation (Fabp3), formation of acetylCoA from citrate (Acly), fatty acid transport (Cpt1b), CoA fatty acid esters (Slc27a2) and increased oxidative phosphorylation (Cox7a1 and Ucp1) are increased by GW501516 in wild-type mice and in untreated PDK1 transgenic mice, but do not correlate with GW501516-induced tumorigenesis. HDAC, histone deacetylase; FA, fatty acid; PDH, pyruvate dehydrogenase; inhibition.

The present study has examined the effect of PDK1 transgene expression on mammary carcinogenesis, and how it impacts the tumor promoting effects of PPARδ activation. Although PDK1 transgenic mice did not exhibit changes in mammary gland development, function and tumorigenicity, they were markedly sensitive to GW501516 treatment, where median tumor-free survival was reduced four-fold in comparison to a two-fold reduction in GW501516-treated wild-type mice. This striking effect correlated with an increase in a specific metabolic gene signature indicative of glycolysis and fatty acid biosynthesis that was not present in either control wild-type or transgenic mice (Figure 5, Supporting information Figure S3). It is well-known that human cancers exhibit a near ubiquitous expression of metabolic genes [45] that is widely regarded to support high rates of proliferation [46]. This phenotype is consistent with the acceleration of mammary tumorigenesis by GW501516 [29], the increase in fatty acid biosynthesis by GW501516 in muscle cells [47], and our analysis of the mammary gland metabolome in GW501516-treated animals. Some of these changes may be related to increased AKT activation in GW501516-treated animals, since it is an important regulator of glucose and lipid metabolism in both normal and malignant cells, including breast cancer [48], [49]. AKT phosphorylates and activates ATP:citrate lyase (Acly) to promote tumor growth [50], and loss of Acly counteracts AKT-driven tumorigenesis [51]. In addition, the PPARδ target gene, Pdk4, [47], [52] reduces the flux of pyruvate into the tricarboxylic acid cycle, and Acss2, which increases the flux of lactate to acetylCoA, were increased in GW501516-treated animals (Table 2, Figure 5). These changes are consistent with the greater levels of fatty acid and phospholipid metabolites in GW501516-treated transgenic mice in comparison to treated wild-type mice (Table 3), suggesting their involvement in enhanced tumorigenesis. This association is also in agreement with the increase in serum lysophospholipids in women with high grade ovarian cancer [53].

The lack of tumorigenicity of PDK1 transgene expression is in agreement with previous studies that found PDK1 over-expression *per se* was not oncogenic unless expressed in a heterozygous PTEN background [54] or together with a growth factor receptor with oncogenic potential, such as erbB2 [12]. The lack of change in pmTOR, p4EBP1, pS6, pPKCα (Figure 1C) and pRSK (results not shown) in mammary tissue from PDK1 transgenic mice is also consistent with the lack of change in S6K and RSK activation following treatment of PDK1 hypomorphic mice with insulin [10]. Thus, low residual levels of PDK1 appear to be sufficient for mammary gland development and function and for downstream signaling.

One seminal finding in our study was the increase in PPARδ expression in the transgenic mammary gland. PPARδ expression is induced by K-Ras via ERK activation [55], and although PDK1 has been reported to regulate MEK1/2 activation [56], ERK activation remained unchanged in MMTV-PDK1 mice (results not shown). Another possible mechanism is that increased pS9GSK3β by AKT inhibits the ability of GSK3β to phosphorylate and destabilize β-catenin by proteasomal degradation, and thus results in enhanced transcription of TCF target genes, such as PPARδ [57]. However, no evidence of nuclear β-catenin accumulation was noted in GW501516-treated wild-type animals despite increased pT308AKT expression (Fig. 3C), suggesting other signaling mechanisms. The changes in PPARδ observed in transgenic mice may also have resulted from post-translational stabilization against proteasomal degradation when ligand-bound [58]. This view is supported by our results in PDK1-transduced mouse mammary epithelial cells, which expressed increased PPARδ and PPARδ-dependent reporter gene activity (Figure 3C). It is equally plausible that endogenous PPARδ ligands generated by fatty acid metabolism serve as PPARδ ligands [59] to increase expression post-translationally. Lastly, the co-association of PDK1 and PPARδ noted in mammary tumors [29] may also have enhanced resistance of the receptor to ubiquitination and degradation.

In addition to increased expression of PPARδ in MMTV-PDK1 mice, PDK1 levels were increased by PPARδ agonist GW501516 (Figure 3B). The mouse PDK1 locus contains a PPRE in an upstream enhancer region [36], and deletion of PPARδ resulted in a 50% reduction in PDK1 mRNA and >80% decrease in PDK1 protein expression [35]. This could result in a feed forward mechanism resulting from the effect of PDK1 and PPARδ on each others expression, and could explain their synergism in tumorigenesis.

Many of the genes whose expression increased more than 3-fold in MMTV-PDK1 mice were associated with muscle architecture or motor function, e.g. myosin, nebulin, troponin 1, tropomyosin and titin. A recent study profiling gene expression in the mammary gland side population also noted a muscle-specific expression pattern [60]. Although the function of these genes in non-myogenic cells is unknown, troponin 1 has been found to bind ERRα, enhance its transcriptional activity [61] and regulate the metabolic switch to oxidative phosphorylation [62]. Thus, the expression of these genes may be a factor in the increase in fatty acid transport and oxidation in muscle cells treated with GW501516 [47].

In summary, PDK1 expression in the mammary gland was not oncogenic, but accelerated tumor formation in conjunction with a PPARδ agonist. GW501516-enhanced tumorigenesis was associated with a distinct gene and metabolomic signature related to glycolysis and fatty acid biosynthesis. These results suggest that PDK1 and PPARδ may drive tumorigenesis by enhancing energy metabolism.

## Supporting Information

Figure S1 (A) Whole mounts of the mammary gland at 3 and 12 weeks of age in founder 192. *Upper panel*, Magnification 5×; *lower panel*, Magnification 20×. (B) Response of PDK1 transgenic mice to lactation and involution. Western blot of PDK1 expression in non-lactating transgenic mice (*+(1)* and *+(2)*) and in lactating mice at day 1 (*D1*) and day 10 (D10) following forced involution by teat sealing. (C) Lactation and involution in transgenic mice. H&E stained sections were prepared on day 1 (*D1*), day 3 (*D3*), day 7 (*D7*) and day 10 (*D10*) of lactating wild-type (−) and transgenic (+) mice following forced involution.
(DOC)

Figure S2 Gene expression profiling of the mammary gland from MMTV-PDK1 and wild-type mice before and after treatment with GW501516. (A) Gene expression in wild-type (*WT*) and MMTV-PDK1 (*PDK1*) mice with and without GW501516 treatment. Untreated MMTV-PDK1 mice expressed a phenotype indicative of wild-type mice treated with GW501516. A list of gene expression changes is included in Table S1. (B) qRT-PCR and gene microarray analysis. Shown are the –fold changes in mammary gene expression between MMTV-PDK1 mice (*PDK1*), PDK1 mice treated with GW510516 (*PDK1+GW*), and wild-type mice treated with GW501516 (*WT+GW*) relative to untreated WT mice. Each experimental group is based on pooled samples from five mice.
(DOC)

Figure S3 PLS-DA scores plots demonstrating clustering of the metabolomic data. Five samples of each group, wild-type (*WT*), MMTV-PDK1 (*PDK1*) and GW501516 (*GW*)-treated WT and PDK1 mice were analyzed by UPLC-ESI-TOFMS and analyzed as described under Materials and Methods. The plot of scores [t1] and [t2] are weighted averages, and the points in the plot are the individual observations of the data for (A) WT vs. PDK1 mice, (B) WT vs. GW-treated WT mice, and (C) PDK1 vs. GW-treated PDK1 mice. Observations near each other in the plot are similar and observations far away from each other are dissimilar.
(DOC)

Figure S4 Gene ontology associated with the heatmap subgroups for MMTV-PDK1 and GW501516-treated mice. The subgroups are listed in Table 2, genes showing a 3-fold or greater changes are listed in Table S1, and gene ontology is listed in Table S2.
(DOC)

Table S1 Gene microarray analysis of control and GW501516-treated animals.
(XLS)

Table S2 Gene ontology of heatmap subgroups.
(XLS)

Table S3 Metabolites in the mammary gland of GW501516-treated wild-type mice vs. wild-type mice.
(XLS)

Table S4 Metabolites in the mammary gland of MMTV-PDK1 mice vs. wild-type mice.
(XLS)

Table S5 Metabolites in the mammary gland of GW501516-treated MMTV-PDK1 mice vs. wild-type mice.
(XLS)
