# Supplementary figures and images for: PPARδ Activation Acts Cooperatively with 3-Phosphoinositide-Dependent Protein Kinase-1 to Enhance Mammary Tumorigenesis
Source: PLoS One. 2011 Jan 13;6(1):e16215. doi: 10.1371/journal.pone.0016215 (PMC3020974; doi:10.1371/journal.pone.0016215)

**A**

**B**

**C**


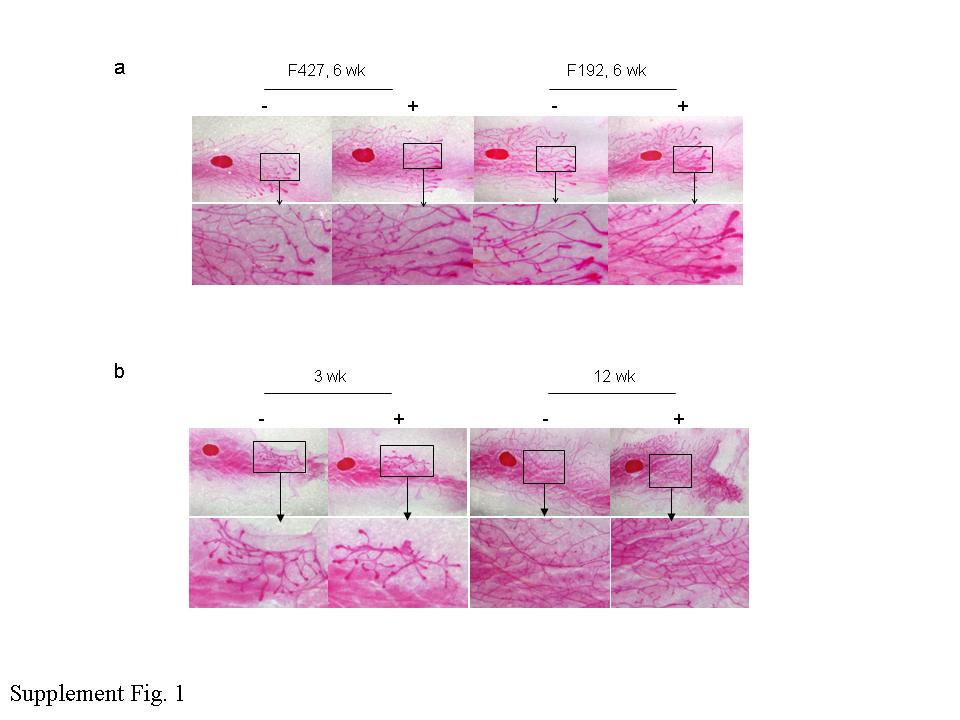

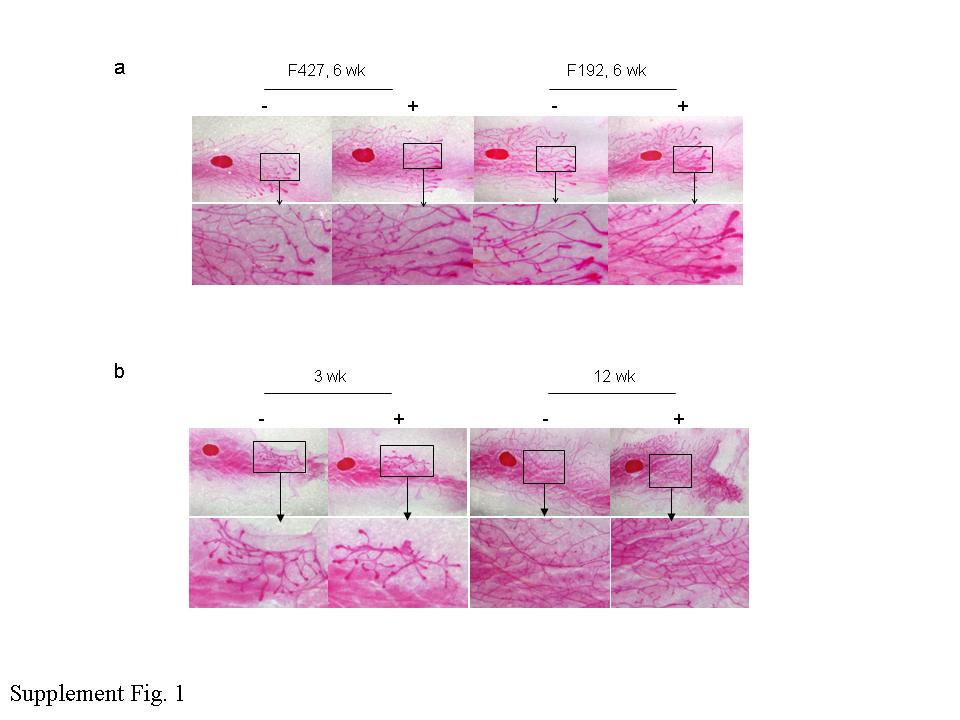


**_________3 wk_________ _________12 wk_________**

**- + - +**

**+(1) +(2) D1 D10**

**PDK1**

**Actin**


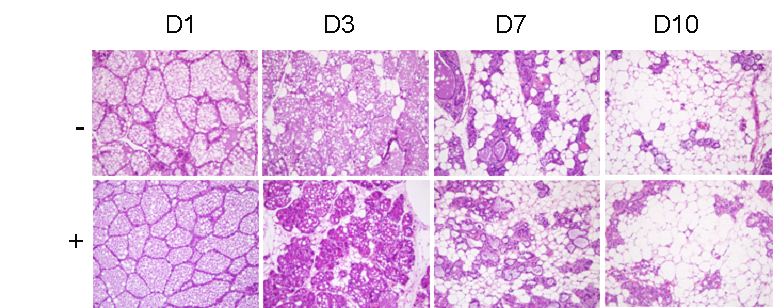


**D1 D3 D7 D10**

**-**

**+**

**Figure S1**

Supplement: Figure S1 — (A) Whole mounts of the mammary gland at 3 and 12 weeks of age in founder 192. Upper panel, Magnification 5×; lower panel, Magnification 20×. (B) Response of PDK1 transgenic mice to lactation and involution. Western blot of PDK1 expression in non-lactating transgenic mice (+(1) and +(2)) and in lactating mice at day 1 (D1) and day 10 (D10) following forced involution by teat sealing. (C) Lactation and involution in transgenic mice. H&E stained sections were prepared on day 1 (D1), day 3 (D3), day 7 (D7) and day 10 (D10) of lactating wild-type (−) and transgenic (+) mice following forced involution. (DOC) [file pone.0016215.s001.doc]

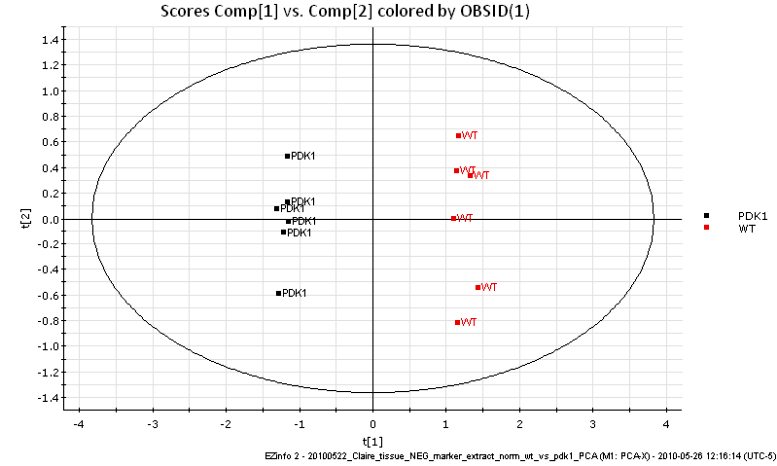


**A**

**B**

**C**


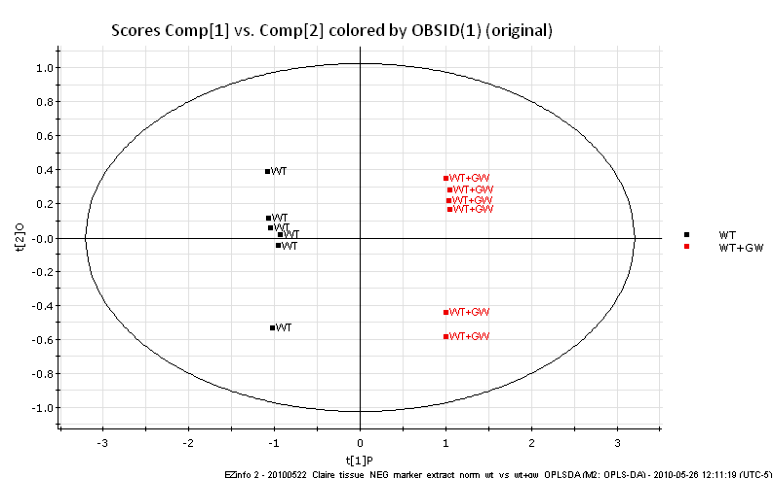


**Figure S3**

Supplement: Figure S3 — PLS-DA scores plots demonstrating clustering of the metabolomic data. Five samples of each group, wild-type (WT), MMTV-PDK1 (PDK1) and GW501516 (GW)-treated WT and PDK1 mice were analyzed by UPLC-ESI-TOFMS and analyzed as described under Materials and Methods. The plot of scores [t1] and [t2] are weighted averages, and the points in the plot are the individual observations of the data for (A) WT vs. PDK1 mice, (B) WT vs. GW-treated WT mice, and (C) PDK1 vs. GW-treated PDK1 mice. Observations near each other in the plot are similar and observations far away from each other are dissimilar. (DOC) [file pone.0016215.s003.doc]

**
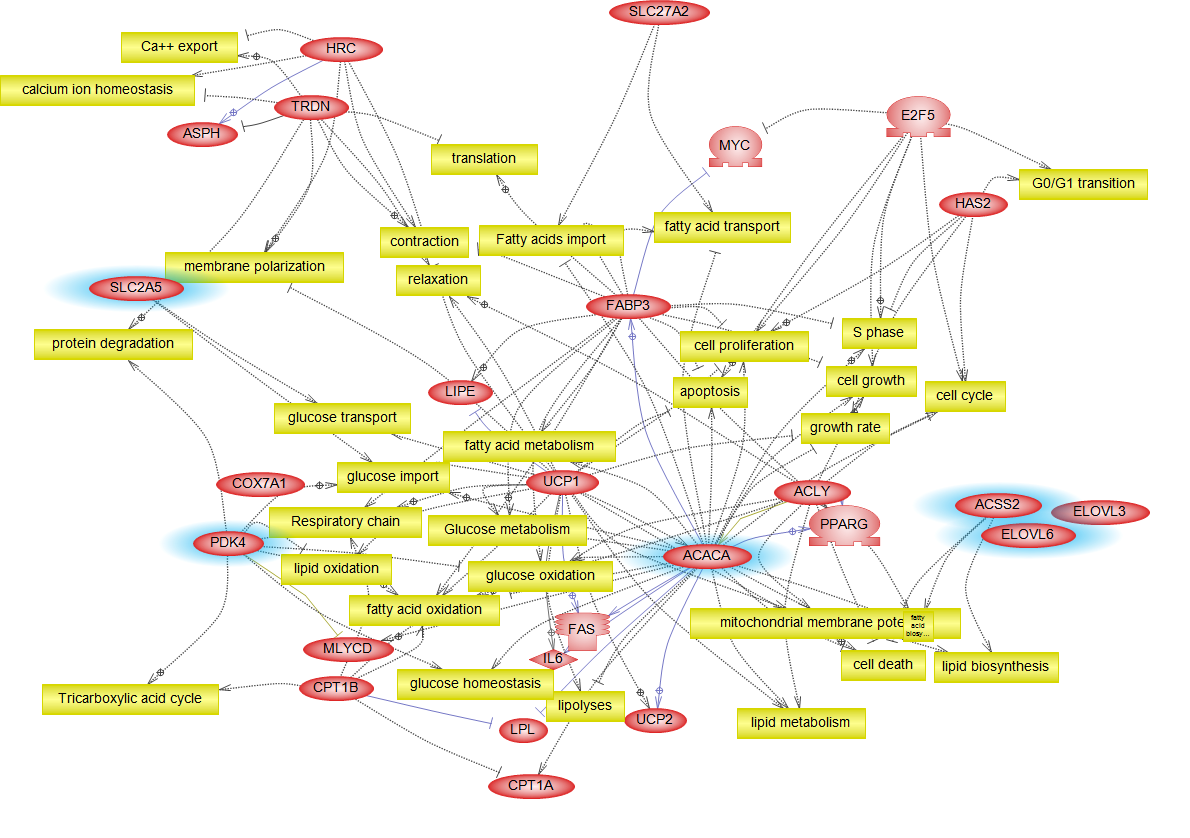
**

**Figure S4**

Supplement: Figure S4 — Gene ontology associated with the heatmap subgroups for MMTV-PDK1 and GW501516-treated mice. The subgroups are listed in Table 2, genes showing a 3-fold or greater changes are listed in Table S1, and gene ontology is listed in Table S2. (DOC) [file pone.0016215.s004.doc]
