# Supplementary material for: PPARδ Activation Acts Cooperatively with 3-Phosphoinositide-Dependent Protein Kinase-1 to Enhance Mammary Tumorigenesis
Source: PLoS One. 2011 Jan 13;6(1):e16215. doi: 10.1371/journal.pone.0016215 (PMC3020974; doi:10.1371/journal.pone.0016215)
Supplement: Figure S2 — Gene expression profiling of the mammary gland from MMTV-PDK1 and wild-type mice before and after treatment with GW501516. (A) Gene expression in wild-type (WT) and MMTV-PDK1 (PDK1) mice with and without GW501516 treatment. Untreated MMTV-PDK1 mice expressed a phenotype indicative of wild-type mice treated with GW501516. A list of gene expression changes is included in Table S1. (B) qRT-PCR and gene microarray analysis. Shown are the –fold changes in mammary gene expression between MMTV-PDK1 mice (PDK1), PDK1 mice treated with GW510516 (PDK1+GW), and wild-type mice treated with GW501516 (WT+GW) relative to untreated WT mice. Each experimental group is based on pooled samples from five mice. (DOC) [file pone.0016215.s002.doc]

**A B**

**B**

**Figure S2**
